# Long-Term Performance of Epicardial versus Transvenous Left Ventricular Leads for Cardiac Resynchronization Therapy

**DOI:** 10.3390/jcm12185766

**Published:** 2023-09-05

**Authors:** Gijs E. de Maat, Bart A. Mulder, Martijn E. Van de Lande, Rajiv S. Rama, Michiel Rienstra, Massimo A. Mariani, Alexander H. Maass, Theo J. Klinkenberg

**Affiliations:** 1 Department of Cardio-Thoracic Surgery, University Medical Center Groningen, P.O. Box 30.001, 9700 RB Groningen, The Netherlands; gijsdemaat@hotmail.com (G.E.d.M.); m.mariani@umcg.nl (M.A.M.); t.j.klinkenberg@umcg.nl (T.J.K.); 2 Department of Cardiology, University Medical Center Groningen, P.O. Box 30.001, 9700 RB Groningen, The Netherlands; b.a.mulder@umcg.nl (B.A.M.); m.e.van.de.lande@umcg.nl (M.E.V.d.L.); r.s.rama@umcg.nl (R.S.R.); m.rienstra@umcg.nl (M.R.)

**Keywords:** cardiac resynchronization therapy (CRT), left ventricular lead, epicardial lead, lead performance, video-assisted thoracic surgery (VATS)

## Abstract

**Aims**: to study the technical performance of epicardial left ventricular (LV) leads placed via video assisted thoracic surgery (VATS), compared to transvenously placed leads for cardiac resynchronization therapy (CRT). **Methods**: From 2001 until 2013, a total of 644 lead placement procedures were performed for CRT. In the case of unsuccessful transvenous LV lead placement, the patient received an epicardial LV lead. Study groups consist of 578 patients with a transvenous LV lead and 66 with an epicardial LV lead. The primary endpoint was LV-lead failure necessitating a replacement or deactivation. The secondary endpoint was energy consumption. **Results**: The mean follow up was 5.9 years (epicardial: 5.5 ± 3.1, transvenous: 5.9 ± 3.5). Transvenous leads failed significantly more frequently than epicardial leads with a total of 66 (11%) in the transvenous leads group vs. 2 (3%) in the epicardial lead group (*p* = 0.037). Lead energy consumption was not significantly different between groups. **Conclusions**: Epicardial lead placement is feasible, safe and shows excellent long-term performance compared to transvenous leads. Epicardial lead placement should be considered when primary transvenous lead placement fails or as a primary lead placement strategy in challenging cases.

## 1. Introduction

Cardiac resynchronization therapy (CRT) has evolved as an effective method for treating patients with systolic heart failure and conduction delay refractory to pharmacological treatment. CRT has been shown to reduce morbidity and mortality in these patients [1,2]. Left ventricular (LV) leads are normally placed/positioned in one of the venous side branches, accessed via the coronary sinus [1,2]. However, transvenous LV pacing is unsuccessful in up to 10% of patients due to unstable lead positions, high thresholds, intractable phrenic nerve stimulation, and unsuccessful placement due to (venous) anatomical reasons [3,4,5]. For patients in which transvenous implantation fails, a different modality for LV stimulation is needed. Alternative routes for LV pacing include intraventricular pacing via the interatrial septum [6] or transapically [7], but surgical epicardial lead placement is the most commonly used technique [8]. Retrospective and prospective studies have shown that epicardial LV lead placement is both feasible and safe [9,10,11,12,13]. When analyzing LV lead performance, it is important to assess not only how many leads have to be replaced but also how many had been terminated due to intractable phrenic nerve stimulation or high thresholds. Furthermore, LV lead energy consumption is important as it is often the major determinant of battery longevity of CRT devices. 

The aims of this study are to analyze epicardial and transvenous lead performance, and to compare energy consumption and all-cause lead failure between both LV leads in a large single-center real-life CRT population.

## 2. Materials and Methods

### 2.1. Patients

In this retrospective study, 644 consecutive patients who received a CRT-device in the period from January 2001 until December 2013 were included. Of these patients, 578 patients received a transvenous lead and 66 received an epicardial lead. Eligibility criteria for CRT implantation were based on standard European Society of Cardiology (ESC) guidelines at the time of implantation. In brief, patients had NYHA III/IV despite optimal medical therapy, wide QRS complex, significant QRS prolongation (>150 ms) and LVEF < 35%. Also, patients who have LVEF < 50% and suffer from AV block that are expected to require a high percentage of ventricular pacing were included. All patients were discussed in multidisciplinary consultation and provided informed consent for the procedure.

### 2.2. Transvenous Lead Placement

Placement of transvenous leads took place under local anesthesia. After placing right atrial and right ventricular leads, the coronary sinus was located and catheterized. A venogram was obtained using a balloon catheter after which the LV lead was inserted, preferably into one of the (postero-)lateral veins. The choice for the CRT device model was at the physician’s discretion; different kind of bipolar leads were used (Medtronic (60%), Guidant (19%), Biotronic (19%), St. Jude (1%) and “other” (1%)). When the operator was unable to place the LV lead using transvenous access because of inaccessible anatomy of the coronary sinus, in case of unstable lead position, or phrenic nerve stimulation, patients were referred for thoracoscopic epicardial lead placement. The techniques used to judge good lead position have evolved in recent years, but the most used were RV-LV electrical delay during normal conduction or delay between onset of the QRS complex on surface-ECGs and LV sensing on intracardiac ECGs.

### 2.3. Epicardial Left Ventricular Lead Placement

Left ventricular epicardial leads were placed via video-assisted thoracic surgery (VATS) under general anesthesia and single lung ventilation. During the first stage of the procedure the patient was positioned in the right lateral decubitus position and prepped and draped. Laterally, below the scapula, an incision was made in the seventh or eighth intercostal space for trocar insertion and CO_2_ insufflation. A camera was inserted and after the left lung collapsed, two ports for instrumentation were created under direct vision. The pericardium was opened posterior of the phrenic nerve and at the mid-posterolateral region. An epicardial screw-in lead (Myodex, St. Jude Medical, Minneapolis, MN, USA) was attached to a viable and visible contracting part of the posterolateral myocardium of the LV. Once the lead was attached, pacing thresholds and impedances were assessed. If inadequate, the lead was repositioned until adequate values were obtained. The lead connector was subsequently placed on top of the collapsed left upper lobe. After closure of the incisions and drainage of the pneumothorax, the patient was placed in supine position. The pectoral device pocket was opened and the device was removed from the pocket. The epicardial lead was retrieved via manual palpitation through the second or third intercostal space and connected to the device after which it was repositioned in the pocket and the incision was closed. Our complete technique has been described in detail before [14].

### 2.4. Follow-Up

The initial follow-up moment of both groups was the first day after implantation; thereafter, regular follow up was at 2 and 6 months after implantation, and then every 6 months thereafter. After 36 months, follow up was scheduled yearly, as described previously [15]. Follow-up duration was calculated as the time between implantation to 1 January 2022 or death, whichever came first. All complications of LV leads in the follow-up period were documented, including LV lead failure that was diagnosed at unscheduled follow-up moments. Measured values of the LV lead during follow up were thresholds in Volt (V), impedances in Ohm (Ω) and programmed pulse width in milliseconds (ms). To calculate energy use per delivered pulse we calculated microjoules (microJ) used per beat with the formula Energy (microJ) = [threshold (volts)^2^ × pulse width (ms) × 10^6^]/[impedance (Ohms) × 1000].

### 2.5. Statistical Analysis

Baseline descriptive statistics are presented as means ± standard deviations for continuous variables and counts with percentages for categorical variables. Differences between groups were evaluated by independent sample *t*-tests. Kaplan–Meier curves were used to evaluate lead survival and were analyzed by the log rank test. R’s cumulative incidence analysis was used to take mortality as a competitive risk into account. Kaplan–Meier curves were also used to evaluate dislocation of the leads (using one minus survival). A significance level of <0.05 was assumed to indicate significance. SPSS 28.0.0.1 for Windows was used.

## 3. Results

### 3.1. Patient Population and Baseline Characteristics

Since 2001, 578 consecutive patients with severe heart failure received transvenous CRT implantation at our institution. Since 2005, 66 patients with failed transvenous LV lead implants, due to various reasons (an unstable lead position, phrenic nerve stimulation, an inaccessible coronary sinus or an inability to achieve the optimal lead position), were referred for an epicardial lead placement via VATS. The study population consisted of 644 patients with the majority being male patients, 420 (73%) in the transvenous group and 41 (62%) in the epicardial group (*p* = 0.08). The mean age at implantation was 65.5 ± 11.5 years in the group undergoing transvenous LV lead placement and 63.8 ± 12.2 years in the group undergoing epicardial LV lead placement. The mean follow up was 5.9 ± 3.5 years in the transvenous lead group and 5.5 ± 3.1 years in the epicardial lead group. Patient characteristics are depicted in Table 1.

No re-interventions for bleeding were reported in either patient group. A procedural safety analysis showed one (0.2%) re-intervention for bleeding in the transcatheter group and none for the epicardial group. Acute dislocation was reported in 11 (1.9%) of the transvenous lead group and none in the epicardial group.

### 3.2. Lead Failure

In 66 (11.4%) patients transvenous placement failed, versus in 2 (3%) patients with epicardial placement. In the transvenous group, dislocation was the most frequent reason for lead failure and occurred in 39 (59%) patients, followed by lead fractures in 15 (23%) patients, phrenic nerve stimulations in 7 (10%) patients and infections in 5 (8%) patients (Table 2). Two epicardial leads (3%) failed; one lead was extracted due to infection requiring total system removal and the second lead suffered from malsensing and was therefore deactivated.

**Figure 1:**
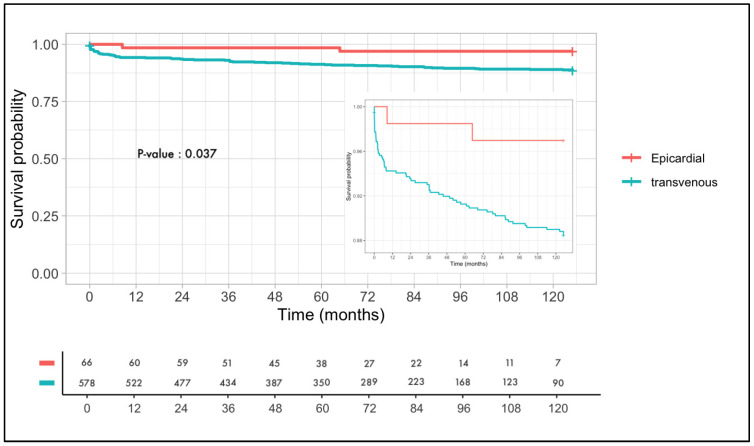
Kaplan–Meier curve of freedom from epicardial and transvenous lead failure.

Figure 1 shows Kaplan–Meier curves of lead survival for all transvenous and epicardial LV leads. Epicardially placed LV leads show a significantly lower rate of lead failure than transvenous placed LV leads with *p* = 0.037. As expected in this patient population, there was a high mortality rate of >50% at 10 years. 

A Kaplan–Meier curve is shown with dysfunction free survival of the LV lead on the *Y*-axis and time in months (from 0 months to 126 months) on the *X*-axis. The epicardial leads are depicted in red and the transvenous leads are depicted in blue. The number at risk is stated for both the epicardial group and the transvenous group. A log rank test is used to show the difference in survival. The log rank is significant with *p* = 0.037 in favor of the epicardial leads.

### 3.3. Electrical Consumption and Stability

**Figure 2:**
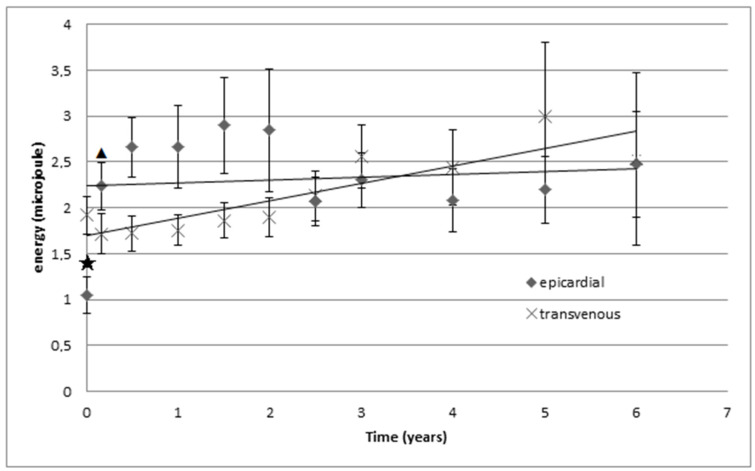
Electrical energy consumption (in micro Joule).

Energy consumption was significantly lower one day after implantation in the epicardial group (*p* = 0.003) but not at other follow-up moments (Figure 2). Energy consumption of epicardial leads was significantly higher at the first follow up than after implantation (*p* = 0.02). When further analyzing the data, there is a trend towards higher energy consumption with epicardial leads during the first years that stabilizes during long-term follow up, whereas transvenous leads show a slow but steady increase in energy consumption (Figure 2), however no statistical significance. 

**Figure 3:**
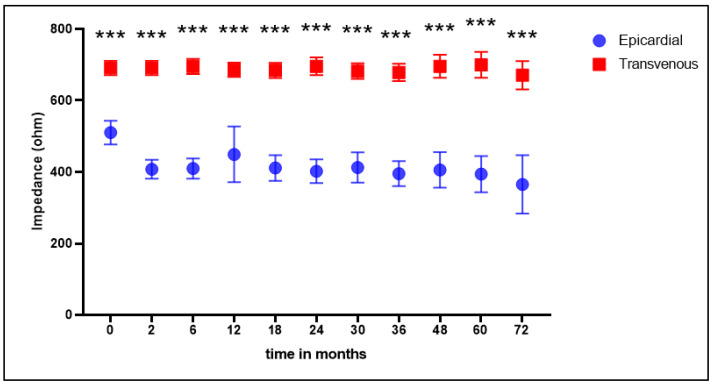
Mean impedance (Ohm). Mean impedance measured in Ohm at baseline and all follow-up moments. Statistically significant differences between groups are marked with *** (*p* < 0.001).

**Figure 4:**
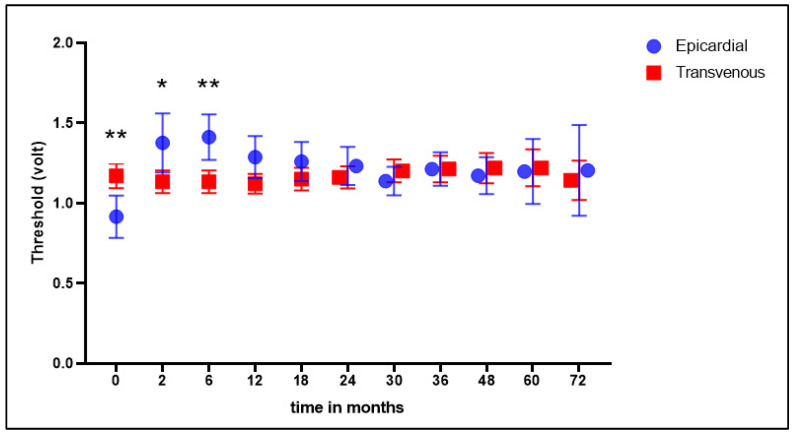
Threshold values (Volt). Mean threshold values measured in Volt at baseline and at all follow-up moments. Statistically significant differences between groups marked with * (*p* < 0.05) or ** (*p* < 0.01).

The mean impedance was significantly higher in the transvenous lead group at baseline and all follow-up moments (Figure 3). The mean threshold was higher at baseline in the transvenous lead group; at 2 and 6 months, this was significantly lower in the transvenous group, and in the following measurements this was not significantly different (Figure 4). 

Electrical parameters (impedance, pulse width and threshold) of transvenous and epicardial LV leads were assessed during follow up and combined with LV lead consumption displayed in micro Joule. Crosses represent transvenous leads and rhombi represent epicardial leads. A star is used to indicate a significant difference in energy consumption between the epicardial LV leads and the transvenous LV leads. A triangle is used to indicate a significant difference in energy consumption to its previous measurement. The epicardial group showed a significantly lower energy consumption in comparison to the transvenous group (*p* = 0.003) at one day after implantation/baseline. Energy consumption increased significantly from baseline to 2 weeks after implantation, in the epicardial group (*p* = 0.02). There were no other notable differences. There is a trend suggesting an increase in energy consumption at long term follow up in the transvenous group.

## 4. Discussion

### 4.1. General 

The present study compared the performance of surgically placed epicardial LV leads in comparison to transvenously placed LV leads for CRT. Furthermore, we studied the incidence and cause of lead failure in our retrospective cohort study. Our study population is comparable with previously reported cohorts [16,17]. In our study, early and late lead failure was significantly more common in transvenous leads than in epicardially placed LV leads and no significant difference in electrical performance was demonstrated during long-term follow up.

### 4.2. Comparison of Epicardial versus Transvenous Placed Leads

There were two earlier studies comparing transvenous and epicardial LV leads. Mair et al. compared the performance of epicardially placed LV leads with transvenous placement in 79 patients. The mean follow up was 16 months. They reported nine lead failures (11%) in the transvenous group and one lead failure (6%) in the epicardial group [9]. Patwala et al. studied 23 patients who underwent epicardial lead placement and compared them to a control group of 35 patients who underwent CRT using the transvenous approach. The follow up was 6 months. Lead failure was reported in one case (4%) of the epicardial group and in one case (3%) of the transvenous group [10]. Both studies showed a low incidence of epicardial lead failure. The same is reported in our study with only two (3%) lead failures in the epicardial group. We confirmed their findings in a large cohort with a long follow-up period. Other trials only assessed epicardial leads without comparing them to transvenous leads. Kamath et al. studied 78 patients who underwent epicardial lead implantation [11]. The mean follow up was 44 months. Lead failure was reported in two cases (3%). The short-term follow up showed a significant increase in pacing thresholds and a decrease in impedance of the leads. The long-term threshold and impedance values remained stable when compared to short-term values. Buiten et al. studied 216 patients who underwent epicardial lead implantation during cardiac surgery [12]. The mean follow up duration was 3 years. Five-year cumulative incidence of lead failure was 2% and 10% for device infection. Both studies showed an excellent performance of epicardial leads with a mean follow up of approximately 3 years. Even though Buiten et al. showed excellent epicardial lead performance, they also reported a significant number of device infections. This might be due to the timing of lead surgery during cardiac surgery. In our study, we have only observed two (3%) lead failures in the epicardial group; in one case due to infection that did not even originate in the epicardial lead but other parts of the system, and in another case the lead was disconnected due to malsensing. These results are comparable to the 5-year result of the large comparative study of Burger et al. [18].

### 4.3. Lead Failure

The most frequent reason of bipolar transvenous lead failure was dislocation. Earlier studies of transvenous lead performance reported early dislocations of the lead (within the first week), but our study also demonstrates 56% (n = 22) late dislocations (after 1 month) of transvenous leads. This has not been previously described in the literature and may suggest that passive fixation of current CS leads is not solid enough or the anatomy of coronary sinus of some patients is not favorable enough for transvenous placement of the LV lead. Another reason for lead failure in seven patients (10%) was intractable phrenic nerve stimulation. The coronary sinus and its tributaries have a variable anatomy and proximity of electrodes with the phrenic nerve can change depending on the patients’ position. In the epicardial group, the anode and cathode of the screw-in lead have no physical interaction with the pericardium making phrenic nerve stimulation very unlikely. Late phrenic nerve stimulation of transvenous leads could be caused by reverse remodeling of LV during follow up. Even though there is no imaging modality proving this point, it is possible that the geometry of the LV changes due to remodeling, possibly rotating the lead closer to the phrenic nerve than during implantation. Lead failure due to phrenic nerve stimulation can be reduced with new developments, e.g., quadripolar leads [19] and active fixation [20], but long-term data on these electrodes are not yet available. Other reasons for lead failure were due to lead fractures in 10 patients (16%) and infections in 6 patients (10%). Lead fractures of epicardial leads have been mentioned in the literature and are often due to mechanical friction with ribs. In our epicardial group, the lead runs through the pleural space to the second or third intercostal space where the lead is picked up via the posterior side of the device pocket [14]. This technique avoids friction of the lead with a hard surface, thereby minimizing the chance of fracture.

### 4.4. Energy Consumption

CRT devices need to be replaced periodically due to battery depletion. This is carried out through a fairly simple surgery under local anesthesia but generator replacement is associated with a high complication rate of up to 4.0% [21]. Since high thresholds of LV leads cause premature battery depletion, this necessitates more frequent replacements compared to single- and dual-chamber pacemakers or ICDs. The observation that during the long term, follow-up energy consumption levels increase at a faster pace for the transvenous placed leads than the epicardial placed leads warrants further investigation.

### 4.5. Epicardial Leads and CRT Response

The question of whether epicardial lead positioning can also lead to improved CRT response has been studied in two small cohorts [22,23] of 9 and 22 patients, respectively. After 6 and 12 months, respectively, they did not find any improvement in echocardiographic parameters or clinical status. A larger sample size and longer follow up could show patients having more benefits from epicardially placed LV leads instead of transvenously placed LV leads. Rackard et al. found no notable difference after a mean follow up of five years in mortality between patients with epicardially placed LV leads or transvenously placed LV leads [24]. The advantage of epicardial lead placement is that there are no anatomic restrictions in placing the LV lead. The STARTER and TARGET trials have shown improved CRT response with LV lead positioning targeted to the area of latest mechanical activation by speckle-tracking echocardiography [25,26]. These trials have shown that even in the targeted groups optimal lead position could not be achieved with transvenous lead placement in a significant proportion of patients. Newer imaging techniques incorporating multi-modality imaging such as MRI and nuclear imaging could even improve identifying the perfect position for the LV lead. Placement of an epicardial LV lead happens under direct vision. This allows the LV lead to be placed at a predetermined point of the last mechanical activation, which is the best place to become a responder of CRT. This fact deserves more recognition and further investigation. After all, it is about becoming a responder. Concomitant epicardial placement of the LV lead during heart surgery could become available for patients with an ejection fraction lower than 30%, but QRS < 120 ms, who are at high risk for needing CRT in the future [27].

### 4.6. Future Perspective

The primary approach to CRT is usually performed by a cardiologist via transvenous delivery of all leads. This procedure is most commonly performed in a cardiac catheterization or electrophysiology laboratory. When transvenous placement of the LV lead is unsuccessful, the procedure is usually aborted and the patient is referred for a second procedure. This usually takes place on a different day, which results in a higher risk of infection due to the re-exploration of the pocket and increased health care costs. In a hybrid setting, a cardio-thoracic surgeon can place the LV lead in the target region followed by transvenous placement of the right-sided electrodes and connection to the device [28]. The potential of such a hybrid procedure for a better response to CRT and lower health care costs has yet to be studied. In recent years, cardiac conduction system pacing has evolved as a technique where CS lead are not possible, and it is also emerging as an alternative to biventricular pacing [29]. This bundle pacing was the initial technique that is being more and more frequently replaced by left bundle branch area pacing (LBBAP) as the latter has been shown to exhibit better ventricular sensing and more stable pacing thresholds [30]. A large observational study demonstrated that the outcomes of LBBAP are comparable to classical biventricular pacing [31]. While conduction system pacing is technically more demanding than standard right ventricular lead placement, a recent consensus paper from EHRA provides guidance in the implantation technique for implanters not yet proficient in this technique [32].

### 4.7. Limitations

The main limitations are the small number of patients included in the epicardial group and the retrospective nature of this study. Because this is a retrospective analysis, it has all the limitations inherent to this type of study, especially selection bias due to the non-randomized layout. Recent developments such as quadripolar leads and active fixation could decrease long-term transvenous lead failure. Furthermore, no data were collected concerning medication use, perioperative parameters, cause of heart failure or response to CRT.

## Figures and Tables

**Table 1:** Patient characteristics.

	Transvenous (N = 578)	Epicardial (N = 66)	*p*-Value
Age, years	65.5 ± 11.5	63.8 ± 12.2	0.13
Male gender, n (%)	420 (73%)	41 (62%)	0.08
Follow up, years	5.9 ± 3.5	5.5 ± 3.1	0.94
Body mass index, kg/m^2^	27 ± 5	29 ± 6	0.12
Diabetes, n (%)	124 (21)	17 (27)	0.30
Coronary artery disease, n (%)	291 (50)	28 (42)	0.19
Hypertension, n (%)	240 (42)	37 (56)	0.02
NYHA class	2.6 ± 0.6	2.5 ± 0.6	0.13
LVEF, %	24.3 ± 8.9	25.6 ± 8.9	0.42
LBBB, n (%)	417 (70)	41 (61)	0.23

Data are displayed as mean ± SD or absolute amount (percentage) unless stated otherwise. NYHA, New York heart association; LVEF, left ventricle ejection fraction; and LBBB, left bundle branch block.

**Table 2:** Lead failure and cause.

	Transvenous (N = 578)	Epicardial (N = 66)
Failure	66 (11%)	2 (3%)
Cause		
Dislocation	39 (59%)	-
Lead fracture	15 (23%)	-
Phrenic nerve stimulation	7 (10%)	-
Infection	5 (8%)	1 (50%)
Malsensing	0 (0%)	1 (50%)

## Data Availability

The data presented in this study are available on request from the corresponding author.
